# Supplementary figures and images for: Yoga pose recognition using dual structure convolutional neural network
Source: PeerJ Comput Sci. 2025 May 27;11:e2907. doi: 10.7717/peerj-cs.2907 (PMC12192719; doi:10.7717/peerj-cs.2907)

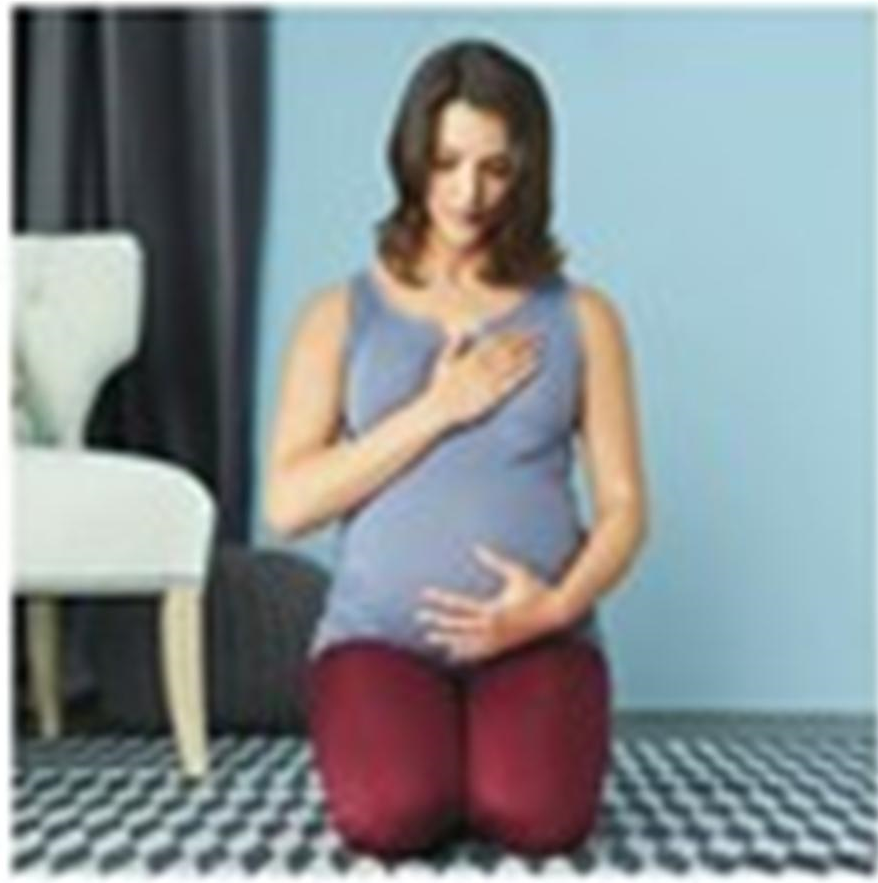

Supplement: Supplemental Information 2 [file peerj-cs-11-2907-s002.zip › datasets1/100 (1).png]

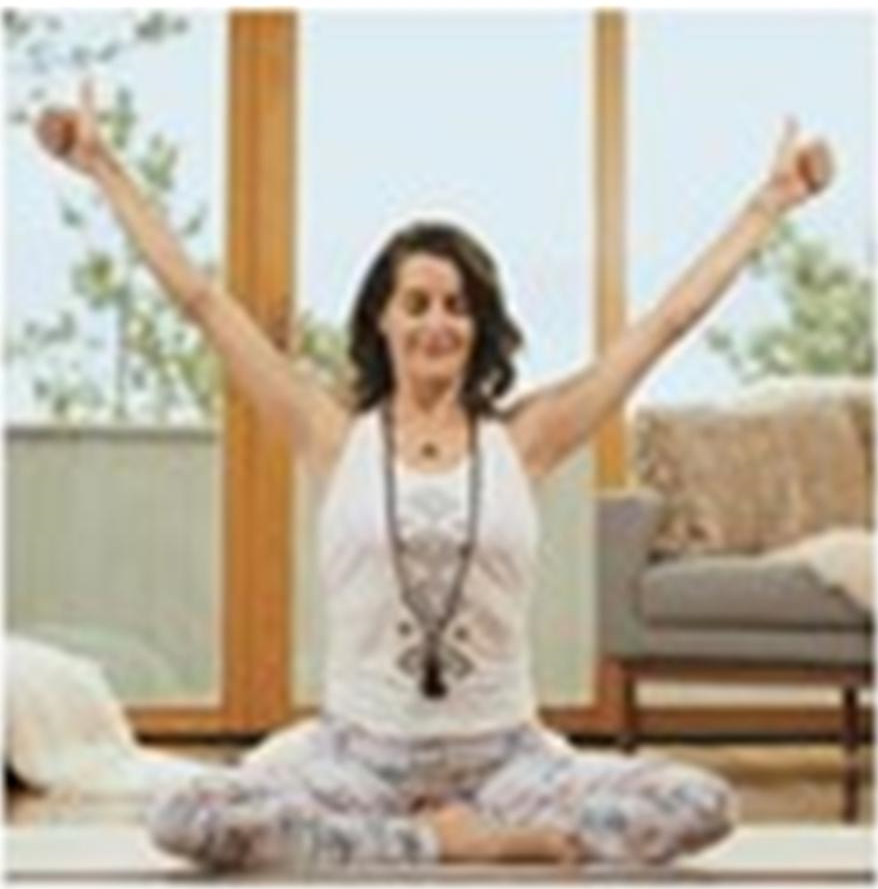

Supplement: Supplemental Information 2 [file peerj-cs-11-2907-s002.zip › datasets1/100 (10).png]

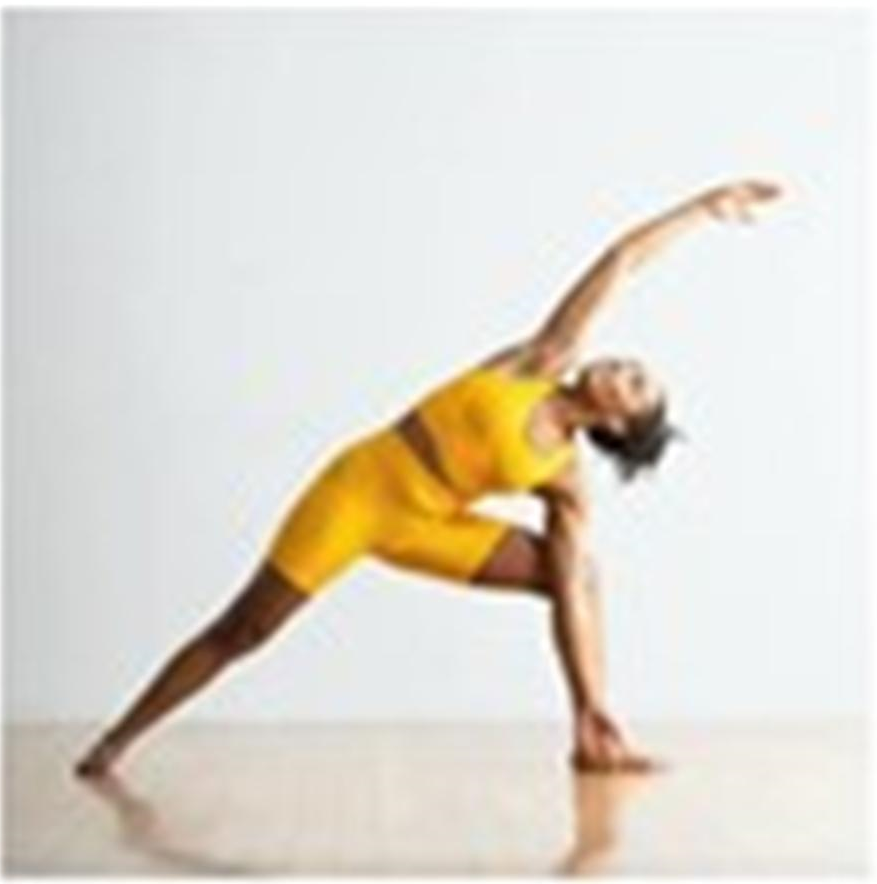

Supplement: Supplemental Information 2 [file peerj-cs-11-2907-s002.zip › datasets1/100 (12).png]

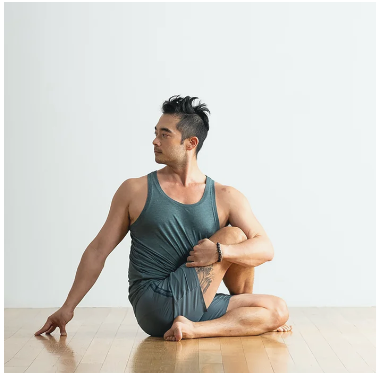

Supplement: Supplemental Information 2 [file peerj-cs-11-2907-s002.zip › datasets1/100 (13).png]

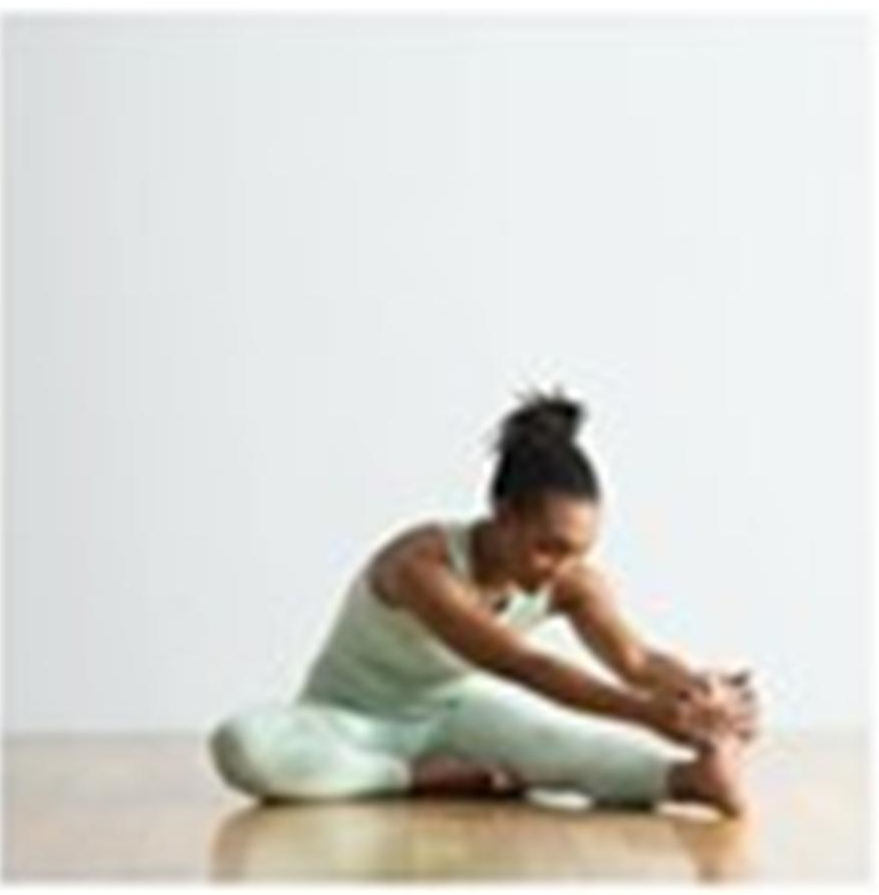

Supplement: Supplemental Information 2 [file peerj-cs-11-2907-s002.zip › datasets1/100 (14).png]
